# Cost-effectiveness analysis of lurbinectedin plus atezolizumab as first-line treatment for extensive-stage small-cell lung cancer

**DOI:** 10.3389/fimmu.2025.1658740

**Published:** 2025-09-23

**Authors:** Yufan Huang, Zheqi Xu, Leilei Bao

**Affiliations:** Department of Pharmacy, Eastern Hepatobiliary Surgery Hospital, Naval Medical University, Shanghai, China

**Keywords:** cost-effectiveness, lurbinectedin, atezolizumab, extensive-stage small-cell lung cancer, partitioned survival model

## Abstract

**Background:**

The IMforte trial demonstrated that lurbinectedin combined with atezolizumab (LU-AT) as a first-line regimen offers clinical advantages over atezolizumab alone (AT) in patients with extensive-stage small-cell lung cancer (ES-SCLC). However, given the high costs of lurbinectedin and atezolizumab, the cost-effectiveness of LU-AT relative to AT remains uncertain. This study aims to assess the cost-effectiveness of LU-AT as a first-line treatment for ES-SCLC within the context of China’s and the United States’ healthcare system.

**Methods:**

A partitioned survival analysis (PartSA) model was employed to assess the cost-effectiveness of LU-AT as a first-line treatment for ES-SCLC. Clinical efficacy data were sourced from the IMforte trial. Drug costs were based on national tender prices, while other costs and utility values were derived from the literature. Outcomes included total costs, quality-adjusted life years (QALYs), and incremental cost-effectiveness ratios (ICERs). One-way sensitivity analysis and probabilistic sensitivity analysis were conducted to assess the robustness of the model.

**Results:**

The combination regimen of lurbinectedin plus atezolizumab yielded an additional 0.21 QALYs compared with atezolizumab monotherapy, leading to an ICER of $374,167.43 per QALY in China and $1,071,237.82 per QALY in the USA, both beyond the willing-to-pay threshold ($40,365.00/QALY in China and $150,000.00/QALY in the USA). The utility of progression-free survival (PFS), the cost of lurbinectedin, body surface area (BSA), and the cost of atezolizumab are the four most influential factors in both China and the United States. Across all sensitivity analyses, the outcomes generated by the models remained robust. At a willingness-to-pay threshold of $40,365 and $150,000 per QALY, the probability of LU-AT being cost-effective relative to AT was 0% in China and USA.

**Conclusion:**

Within the framework of China’s and the United States’ healthcare system, LU-AT is unlikely to represent a cost-effective first-line treatment for ES-SCLC.

## 1 Introduction

Small-cell lung cancer (SCLC) is a highly aggressive malignancy, comprising approximately 15% of all lung cancer cases, with a 5-year survival rate of under 7% (1). Characterized by rapid cell proliferation and early metastasis, it is associated with a poor prognosis. Extensive-stage SCLC (ES-SCLC) accounts for 70% of all SCLC cases (2–4). For decades, etoposide-platinum chemotherapy has been the standard treatment for ES-SCLC. However, while initial response rates are high, most patients develop resistance to the therapy (1). In 2019, immune checkpoint inhibitors (ICIs) revolutionized the treatment landscape for ES-SCLC, with atezolizumab combined with carboplatin and etoposide emerging as the new first-line regimen (5). Despite the improved efficacy of first-line ICI plus platinum-based chemotherapy, most patients eventually relapse, and survival outcomes remain suboptimal.

Currently, the ICIs used for the treatment of extensive-stage small-cell lung cancer (ES-SCLC) mainly include Adebrelimab, benmelstobart, Serplulimab, Atezolizumab, and Durvalumab, among which Atezolizumab and Durvalumab have been recommended as first-line treatment regimens by the National Comprehensive Cancer Network (NCCN) guidelines in the United States and the Guidelines of Chinese society of clinical oncology (CSCO) (6, 7). With the widespread adoption of ICIs in the treatment of ES-SCLC, their economic value has become a global focus of attention. Although these agents can improve survival outcomes, their high pricing often results in incremental cost-effectiveness ratios (ICERs) that exceed local willingness-to-pay (WTP) thresholds. For instance, Atezolizumab and Durvalumab lack cost-effectiveness in both the United States and China (8–10). In addition, other ICIs such as Adebrelimab, benmelstobart, and Serplulimab have been shown to be cost-effective in the United States but not in China (2, 11–13). These discrepancies indicate that characteristics of healthcare systems—such as drug pricing mechanisms, medical insurance reimbursement policies, and clinical practice patterns—exert a significant influence on pharmacoeconomic conclusions.

Lurbinectedin, a synthetic alkylating agent, induces cancer cell death by inhibiting the binding of oncogenic transcription factors to their recognition sequences (14). In a Phase II clinical trial, lurbinectedin monotherapy demonstrated promising results (15). More recently, the Phase III IMforte trial assessed the efficacy and safety of lurbinectedin combined with atezolizumab as first-line maintenance therapy for ES-SCLC (16). The trial showed that the lurbinectedin-atezolizumab combination significantly improved median progression-free survival (PFS; 5.4 months vs. 2.1 months) and median overall survival (13.2 months vs. 10.6 months) compared to atezolizumab alone. These results suggest that lurbinectedin combined with atezolizumab could become a new first-line treatment for ES-SCLC.

Although the IMforte trial demonstrated that LU-AT significantly prolongs survival in patients with ES-SCLC compared to AT (16), its economic feasibility remains unclear, and this research gap urgently needs to be addressed. In current immunotherapy for ES-SCLC, existing regimens such as atezolizumab lack cost-effectiveness in both China and the United States due to high costs (8, 9), while the clinical promotion of new regimens must balance efficacy and economic efficiency. Both Chinese and American healthcare systems require cost evidence to support decision-making. Given that LU-AT has not yet been widely adopted, conducting this evaluation at this stage can prospectively provide a basis for clinical selection and healthcare insurance policy formulation, avoiding resource misallocation or patients missing out on treatment due to financial burdens, which holds important practical significance. Therefore, this study evaluates the cost-effectiveness of lurbinectedin combined with atezolizumab as a first-line treatment for ES-SCLC from the perspective of China’s and the United States’ healthcare system. The primary goal is to provide evidence for treatment decision-making in patients with ES-SCLC. Additionally, the study aims to inform national health insurance policies and support the rational allocation of healthcare resources.

## 2 Methods

### 2.1 Modeling

**Figure 1:**
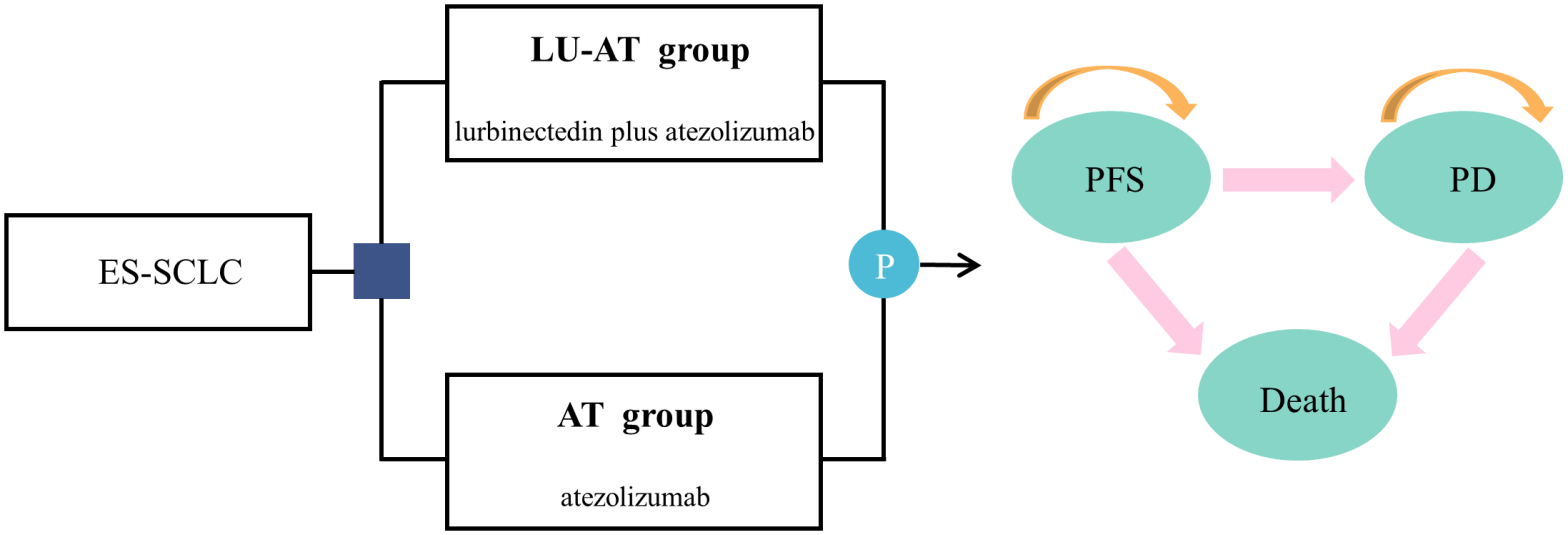
The partitioned survival model simulating outcomes for the IMforte trial. All patients started with PFS state and received treatment with LU-AT or AT. LU-AT, Lurbinectedin-Atezolizumab group; AT, Atezolizumab group; PFS, progression-free survival; PD, progressive disease.

The Partitioned Survival Analysis (PartSA) model in TreeAge2022 software was employed to assess the cost-effectiveness of LU-AT (lurbinectedin combined with atezolizumab) versus AT (atezolizumab monotherapy) as a first-line treatment for ES-SCLC (**Figure 1**). The model incorporated three health states: PFS, disease progression (PD), and death. All patients initially entered the PFS state, with death as the absorbing state. The model’s cycle duration was set at 21 days, totaling 102 cycles over 8.5 years, with 99% of patients projected to die during this period. Model outcomes included total costs, QALYs, and ICERs. In accordance with the Chinese Pharmacoeconomic Evaluation Guidelines, the WTP threshold was set at three times the 2024 per capita GDP of China, amounting to US$40,365 per QALY (17). In contrast, the WTP threshold in the United States is $150,000 per QALY.A treatment strategy is deemed cost-effective if the ICER falls below this threshold.

### 2.2 Patient clinical treatment data

Clinical treatment data were derived from the IMforte trial, a randomized, multicenter, open-label, Phase 3 trial conducted across 96 hospitals and medical centers in 13 countries/regions (Belgium, Germany, Greece, Hungary, Italy, Mexico, Poland, South Korea, Spain, Taiwan, Türkiye, the UK, and the USA) (16). The trial compared lurbinectedin combined with atezolizumab to atezolizumab monotherapy as maintenance therapy for patients with ES-SCLC following standard first-line induction therapy with atezolizumab, carboplatin, and etoposide. Eligible patients were aged ≥ 18 years, had treatment-naive ES-SCLC, and received four cycles of 21-day induction therapy (atezolizumab, carboplatin, and etoposide). After induction, patients were randomized to receive either lurbinectedin (3.2 mg/m^2^) plus atezolizumab (1200 mg) or atezolizumab (1200 mg) intravenously every 3 weeks until PD (per RECIST v1.1), unacceptable toxicity, or withdrawal of consent. Treatment beyond PD was not permitted per the protocol. Unless contraindicated, patients receiving lurbinectedin plus atezolizumab also received prophylactic granulocyte colony-stimulating factor (G-CSF) and antiemetic premedication according to institutional guidelines. Between Nov 17, 2021, and Jan 11, 2024, 895 patients were screened for enrolment, of whom 660 (74%) were enrolled into the induction phase. Between May 24, 2022, and April 30, 2024, 483 (73%) of 660 patients entered the maintenance phase and were randomly assigned to lurbinectedin plus atezolizumab (n=242) or atezolizumab (n=241). According to the trial results, the median duration of treatment was 4.2 months for the LU-AT group and 2.1 months for the AT group, with a median of 7 doses for LU-AT and 4 doses for AT. Based on the second-line treatment data from the IMforte trial, we assumed that 37% of patients in the LU-AT group and 49% of patients in the AT group received chemotherapy after disease progression or the occurrence of unacceptable toxic reactions. In accordance with the NCCN and CSCO guidelines, topotecan-based chemotherapy is recommended as the standard second-line treatment for both groups (6, 7). Since the IMforte trial did not provide data on the duration of second-line treatment, we referred to the trial data by Song et al. and set the maintenance duration of second-line treatment at 1.73 months (18). The remaining patients all received best supportive care (BSC), including palliative radiotherapy, symptom control, nutritional support, and psychological support.

### 2.3 Survival transition probabilities

Kaplan-Meier curves from the IMforte trial were digitized using the GetData Graph Digitizer software. Various survival distributions were then fitted to the reconstructed individual patient data using R software to generate survival curves extending beyond the follow-up period reported in the trial (19). The distributions tested included exponential, gamma, generalized F, generalized gamma, Gompertz, Weibull, log-logistic, and log-normal models (**Supplementary Table B**) (20, 21). Based on the Akaike and Bayesian information criteria (22, 23), the log-logistic distribution was identified as the most suitable model for the original survival curves (**Supplementary Figure 1**; **Table 1**). This approach enabled the estimation of transition probabilities between different health states.

**Table 1:** Relevant parameters of survival distribution.

Variable	Value	Range		Source
		Min	Max	
Survival model for the overall population				
Log-logistic survival model of PFS				
LU-AT group	Scale = 0.1972995	0.1578396	0.2367594	(16)
	Shape = 1.74213	1.393704	2.090556	(16)
AT group	Scale = 0.3984715	0.318777	0.478166	(16)
	Shape = 1.88024	1.504192	2.256288	(16)
Log-logistic survival model of OS				
LU-AT group	Scale = 0.07237747	0.057902	0.086853	(16)
	Shape = 1.800347	1.440278	2.160416	(16)
AT group	Scale = 0.09175307	0.073402	0.110104	(16)
	Shape = 1.844456	1.475565	2.213347	(16)

PFS, progression-free survival; OS, overall survival; LU-AT, Lurbinectedin-Atezolizumab group; AT, Atezolizumab group.

### 2.4 Cost and utility

The study focused exclusively on direct medical costs, including those for medications, tests, routine follow-ups, BSC, management of grade ≥ 3 adverse events with an incidence exceeding 5%, and hospice care (**Table 2**). Drug costs were sourced from national tender prices, while other expenditures were derived from the literature and adjusted to 2024 values using the medical price index from the National Bureau of Statistics of China. The prices in the United States are sourced from Drugs.com (https://www.drugs.com/). All costs were expressed in US dollars, converted at the average 2024 exchange rate (1 USD = 7.12 CNY). As the IMforte trial did not provide quality-of-life data, utility values for PFS and PD were obtained from published studies (2). To mitigate potential bias from using identical utility values for both LU-AT and AT groups, the disutility of grade 3 or higher adverse events with an incidence exceeding 5% in each treatment arm was incorporated, improving the accuracy of health utility values for each group. In compliance with pharmacoeconomic guidelines, all costs and utility values were discounted at an annual rate of 5% (19).

**Table 2:** Basic parameters of the input model and the range of sensitivity analyses.

Variable	Base Value	Range		Distribution	Source
		Min	Max		
LU-AT group: Incidence of AEs (%)					
Anaemia	8	6.4	9.6	Beta	(16)
Decreased neutrophil count	7	5.6	8.4	Beta	(16)
Decreased platelet count	7	5.6	8.4	Beta	(16)
Neutropenia	5	4	6	Beta	(16)
Thrombocytopenia	5	4	6	Beta	(16)
AT group: Incidence of AEs (%)					
Anaemia	2	1.6	2.4	Beta	(16)
Decreased neutrophil count	0	0	0	Beta	(16)
Decreased platelet count	0	0	0	Beta	(16)
Neutropenia	0.42	0.336	0.504	Beta	(16)
Thrombocytopenia	0.42	0.336	0.504	Beta	(16)
Costs ($)(China)					
lurbinectedin (4mg)	2,857.44	2,285.95	3,428.93	Gamma	(24)
atezolizumab (200mg)	4,606.74	3,685.39	5,528.09	Gamma	(24)
Carboplatin (100mg)	5.79	4.632	6.948	Gamma	(24)
Etoposide(100mg)	2.52	2.016	3.024	Gamma	(24)
G-CSF(3mg)	95.51	76.41	114.61	Gamma	(24)
Palonosetron(0.25mg)	2.11	1.69	2.53	Gamma	(24)
Topotecan(2mg)	14.41	11.53	17.30	Gamma	(24)
Best supportive care per cycle	182.97	146.38	219.56	Gamma	(2)
Routine follow-up per cycle	74.05	59.24	88.86	Gamma	(2)
Tests per cycle	358.82	287.056	430.584	Gamma	(2)
Terminal care in end-of-life	1,495.49	1,196.39	1,794.59	Gamma	(2)
Anaemia	104.81	83.85	125.77	Gamma	(2)
Decreased neutrophil count	115.01	92.01	138.01	Gamma	(25)
Decreased platelet count	1,505.92	1,204.74	1,807.10	Gamma	(25)
Neutropenia	83.67	66.94	100.40	Gamma	(2)
Thrombocytopenia	1,083.66	8,66.93	1,300.39	Gamma	(2)
Costs ($)(USA)					
lurbinectedin (4mg)	8,066.50	6453.2	9679.8	Gamma	(26)
atezolizumab (1200mg)	11,328.39	9062.71	13594.07	Gamma	(26)
Carboplatin(100mg)	16.4	13.12	19.68	Gamma	(26)
Etoposide(100mg)	8.43	6.744	10.116	Gamma	(26)
G-CSF(3mg)	361	288.8	433.2	Gamma	(26)
Palonosetron(0.25mg)	9.65	7.72	11.58	Gamma	(26)
Topotecan(4mg)	122.07	97.66	146.48	Gamma	(26)
Best supportive care per cycle	1676.06	1340.85	2011.27	Gamma	(11)
Routine follow-up per cycle	63.43	50.74	76.12	Gamma	(27)
Tests per cycle	661.80	529.44	794.16	Gamma	(10)
Terminal care in end-of-life	39754.07	31803.26	47704.88	Gamma	(28)
Anaemia	9659.74	7727.79	11591.67	Gamma	(10)
Decreased neutrophil count	39992.55	31994.04	47991.06	Gamma	(10)
Decreased platelet count	16371.11	13096.88	19645.33	Gamma	(28)
Neutropenia	17059.51	13647.61	20471.41	Gamma	(28)
Thrombocytopenia	6434.76	5147.80	7721.71	Gamma	(10)
Utility value					
PFS	0.673	0.5384	0.8076	Beta	(2)
PD	0.473	0.3784	0.5676	Beta	(2)
Utility decrement					
Anaemia	0.073	0.058	0.088	Beta	(25)
Decreased neutrophil count	0.2	0.16	0.24	Beta	(25)
Decreased platelet count	0.05	0.04	0.06	Beta	(25)
Neutropenia	0.09	0.072	0.108	Beta	(29)
Thrombocytopenia	0.19	0.152	0.228	Beta	(30)
Other					
Body surface area (m^2^) (China)	1.72	1.38	2.06	Normal	(2)
Body surface area (m^2^) (USA)	1.82	1.6	2.04	Normal	(31)
Discount rate	0.05	0.00	0.08	Fixed	(2)

AE, adverse event; PD, progressive disease; PFS, progression-free survival; OS, overall survival; G-CSF, granulocyte colony-stimulating factor; LU-AT, Lurbinectedin-Atezolizumab group; AT, Atezolizumab group.

### 2.5 Sensitivity analysis

One-way sensitivity analysis and probabilistic sensitivity analysis were performed to evaluate the robustness of the model. In the one-way sensitivity analysis, variables were adjusted within ranges reported in the literature; in the absence of data, variations of ± 20% from the base values were applied. The discount rate was varied from 0% to 8% (**Table 2**). The results were visualized using tornado diagrams. To assess the combined impact of parameter uncertainties, probabilistic sensitivity analysis was conducted through 1000 iterations of Monte Carlo simulations, with each parameter assigned a specific probability distribution (**Table 2**). The results were visualized as scatter plots.

## 3 Results

### 3.1 Basic analysis results

The results of this study are summarized in terms of total costs, QALYs, and ICERs (**Table 3**). The LU-AT group achieved 0.88 QALYs, compared with 0.67 QALYs in the AT group, resulting in an incremental gain of 0.21 QALYs. In China, the total cost was US$134,590.58 for the LU-AT group and US$55,632.29 for the AT group. This led to an incremental cost of US$78,958.29 and an ICER of US$374,167.43 per additional QALY gained. In the United States, total costs were US$400,754.83 and US$174,698.00 for LU-AT and AT, respectively, corresponding to an incremental cost of US$226,056.83 and an ICER of US$1,071,237.82 per incremental QALY. Both ICERs exceed the WTP thresholds, indicating that first-line LU- AT for ES-SCLC is unlikely to represent a cost-effective strategy from either the Chinese or the US perspective.

**Table 3:** The cost and outcome results of the cost-effectiveness analysis.

Country	Regimen	LU-AT	AT	Incremental
China	Total QALYs	0.88	0.67	0.21
	Total costs, $	134,590.58	55,632.29	78,958.29
	ICER, $ Per QALY			374,167.43
USA	Total QALYs	0.88	0.67	0.21
	Total costs, $	400,754.83	174,698.00	226,056.83
	ICER, $ Per QALY			1,071,237.82

ICER, incremental cost-effectiveness ratio; LU-AT, Lurbinectedin-Atezolizumab group; AT, Atezolizumab group; QALY, quality-adjusted life year.

### 3.2 Sensitivity analysis

**Figure 2:**
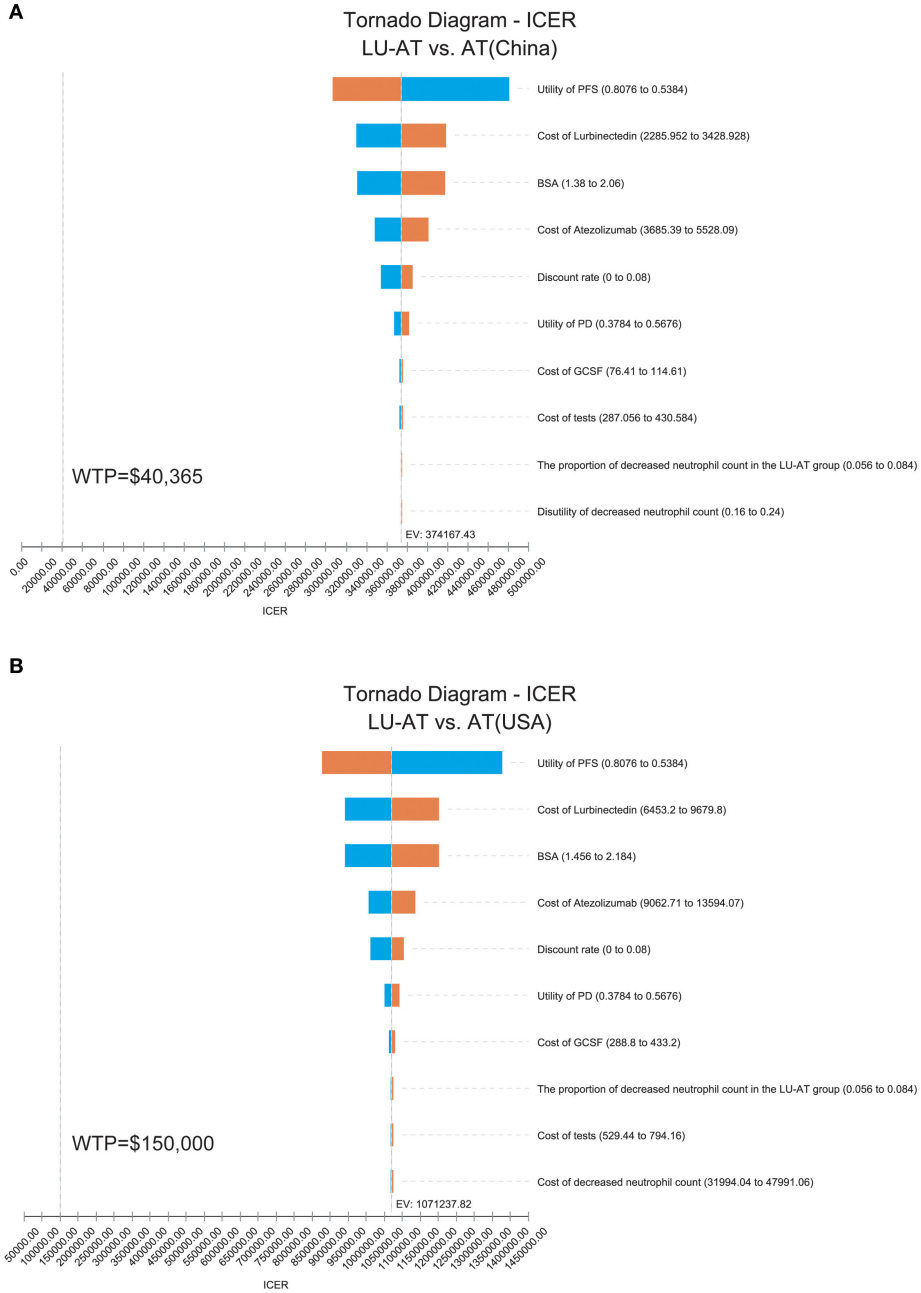
One-way sensitivity analyses in the overall population. The tornado diagram of one-way sensitivity analysis in China **(A)**; The tornado diagram of one-way sensitivity analysis in the United States **(B)**; ICER, incremental cost-effectiveness ratio; LU-AT, Lurbinectedin-Atezolizumab group; AT, Atezolizumab group; PFS, progression-free survival; PD, progressive disease; WTP, willingness-to-pay.

**Figure 3:**
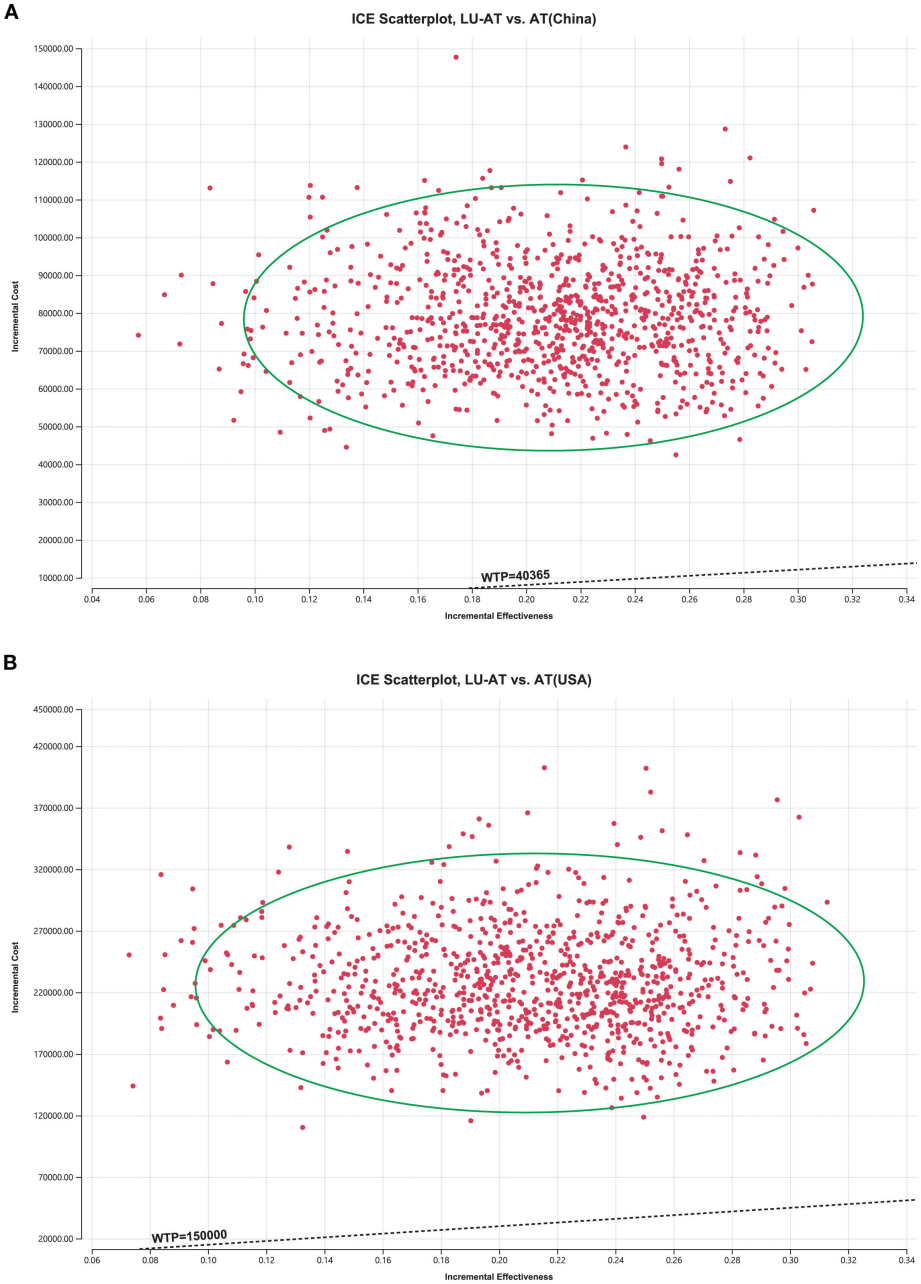
The cost-effectiveness probabilistic scatter plot in China **(A)**; The cost-effectiveness probabilistic scatter plot in the United States **(B)**; Ellipses are used to indicate 95% confidence intervals. Points that lie below the ICER threshold represent cost-effective simulations. LU-AT, Lurbinectedin-Atezolizumab group; AT, Atezolizumab group; PFS, progression-free survival; PD, progressive disease; WTP, willingness-to-pay.

The results of the one-way sensitivity analysis are presented in the form of a tornado diagram (**Figure 2**). The most influential parameters on the model were the utility of PFS, the cost of lurbinectedin, BSA, and the cost of atezolizumab. When these parameters were allowed to vary within their specified ranges, the ICER consistently exceeded the predefined WTP threshold, suggesting that variations in input parameters did not significantly alter the model’s outcome and indicating the robustness of the results. The results of the probabilistic sensitivity analysis are shown as a scatter plot (China: 95% CI:270324.33–488936.48; USA: 95% CI:786393.67–1446803.88) (**Figure 3**). At the WTP threshold of $40,365 and $150,000 per QALY, the probability of LU-AT being cost-effective compared to AT was 0%. Even when the price of lurbinectedin was reduced to zero, LU-AT still did not prove to be cost-effective, likely due to the high costs associated with AT, which were exacerbated by the extended survival observed in the LU-AT group.

### 3.3 Scenario analysis

In Scenario 1, where the modeling duration was adjusted to 2, 4, and 6 years, the ICERs for LU-AT compared to AT were $528,658.82 per QALY, $417,303.22 per QALY, and $386,150.68 per QALY, respectively (**Table 4**). In Scenario 2, to eliminate the impact of ethnic factors, we conducted a sensitivity analysis on the survival curve parameters. The results showed that this did not alter the conclusion that LU-AT lacks cost-effectiveness.

**Table 4:** Scenario analyses in overall population.

Country	Scenarios	Cost ($)		QALY		ICER ($/QALY)
		LU-AT group	AT group	LU-AT group	AT group	
China	Model runtime (year) =2	117,146.44	52,040.10	0.65	0.53	535,117.06
	Model runtime (year) =4	128,221.46	54,377.09	0.80	0.62	422,205.32
	Model runtime (year) =6	132,235.74	55,179.45	0.85	0.65	390,617.49
USA	Model runtime (year) =2	352,821.85	165,045.01	0.65	0.53	1,543,360.99
	Model runtime (year) =4	383,232.20	171,311.63	0.80	0.62	1,211,656.26
	Model runtime (year) =6	394,275.41	220,800.58	0.85	0.65	1,119,293.04

ICER, incremental cost-effectiveness ratio; LU-AT, Lurbinectedin-Atezolizumab group; AT, Atezolizumab group; BSC, best supportive care.

## 4 Discussion

The IMforte trial evaluated the safety and efficacy of LU-AT versus AT as maintenance therapy for patients with ES-SCLC following standard first-line induction therapy with atezolizumab, carboplatin, and etoposide (16). The trial results demonstrated that LU-AT significantly prolonged median PFS (5.4 months vs. 2.1 months) and median overall survival (13.2 months vs. 10.6 months) compared to the AT group. These findings suggest that lurbinectedin combined with atezolizumab may serve as a novel first-line treatment option for patients with ES-SCLC. However, the high cost of LU-AT could limit its widespread adoption, particularly among patients with financial constraints. Therefore, the primary objective of this study was to assess the cost-effectiveness of LU-AT as a first-line treatment strategy for ES-SCLC within the Chinese and the United States’ healthcare system. The analytical results showed that in China, the incremental cost per QALY gained with LU-AT was $374,167.43, while in the United States, the incremental cost per QALY gained with LU-AT reached $1,071,237.82. The incremental costs in both China and the United States significantly exceeded the WTP thresholds. Therefore, LU-AT is deemed not cost-effective as a first-line treatment for ES-SCLC in both China and the United States.

The lack of cost-effectiveness of the LU-AT regimen is attributed to the requirement for long-term maintenance therapy with both lurbinectedin and atezolizumab, which substantially increases overall treatment costs without providing sufficient incremental survival benefits. However, these results should not be interpreted as a rationale to restrict the use of LU-AT, as this could potentially deprive patients of valuable therapeutic opportunities. One-way sensitivity analysis identified the cost of lurbinectedin as a key determinant of the model’s outcomes. Even when the cost of lurbinectedin was reduced to zero, the LU-AT regimen remained non-cost-effective. This was likely due to the prolonged administration of atezolizumab in the LU-AT group, which extended treatment duration without delivering proportional clinical benefits, despite modest survival gains. Meanwhile, scenario analysis—a methodology used to evaluate the cost-effectiveness of pharmaceuticals by incorporating various hypothetical conditions and uncertain factors to better reflect real-world circumstances—demonstrated that extending the treatment cycle could enhance the cost-effectiveness of LU-AT. This suggests that improving treatment adherence can optimize therapeutic value, which aligns with the interests of clinicians, patients, and their families, as well as broader ethical and social considerations.

Numerous anti-tumor drugs are deemed economically inefficient due to their inability to achieve favorable ICERs, such as benmelstobart combined with anlotinib (2) and adebrelimab combined with chemotherapy (2), which aligns with the findings of this study. Since the establishment of the National Health Commission of China in 2018, the country has initiated several rounds of drug price negotiations with pharmaceutical companies through national procurement strategies aimed at alleviating the financial burden on cancer individuals. Thus, the prices of many anti-cancer drugs have decreased by 30% to 70% (32). In tertiary hospitals, the reimbursement rate for insured patients’ medical expenses is approximately 70%, with primary healthcare institutions typically offering even higher reimbursement rates (33). With the progression of national medical insurance price negotiations in China, several treatment regimens have become cost-effective. For example, Yang et al. reported that Toripalimab combined with chemotherapy may represent a cost-effective first-line treatment for ES-SCLC (25), while Long et al. found that Tislelizumab plus chemotherapy could be the preferred option for patients with ES-SCLC (34). These improvements are likely attributed to reductions in drug costs following China’s national medical insurance volume-based procurement. The price of Toripalimab has dropped from $383.63 in 2021 to $261 in 2024 (25, 35), while Tislelizumab’s price has decreased from $675.84 in 2022 to $176.06 in 2025 (36). Meanwhile, the United States is also actively exploring measures to regulate drug prices. In 2025, the U.S. government took targeted actions to address the issue of exorbitant drug prices: on May 12 local time, President Donald Trump signed an executive order adopting the “most-favored-nation” principle (37). It mandates the U.S. Department of Health and Human Services to formulate an OECD-aligned “most-favored-nation price target” within 30 days, anchoring U.S. drug prices to the lowest levels among OECD member countries to tackle the prevalence of generally higher drug prices in the United States. This policy covers all prescription drugs and focuses on medications with high expenditure and significant price disparities, such as weight-loss drugs and chronic disease medications.

The findings of this study provide direct evidence to inform national price negotiations and potential healthcare insurance access decisions for LU-AT, covering perspectives from both China’s and the United States’ healthcare systems. In China, where cost-effectiveness is increasingly emphasized in the evaluation of the National Reimbursement Drug List (NRDL), this study shows that the current ICER of LU-AT ($374,167.43 per QALY) far exceeds the WTP threshold of $40,365 per QALY, indicating that its pricing is incompatible with the affordability of the healthcare system. This provides critical evidence for policymakers: substantial price reductions for lurbinectedin or atezolizumab through negotiations are necessary to bring the ICER below the threshold, following the successful examples of anti-tumor drugs such as toripalimab and tislelizumab (25, 35, 36), which improved cost-effectiveness and gained NRDL inclusion through negotiated price cuts. In the United States, based on the WTP threshold of $150,000 per QALY, LU-AT’s ICER ($1,071,237.82 per QALY) also significantly exceeds the threshold. This offers insights for U.S. reimbursement decision-making: at the current pricing, LU-AT is unlikely to secure healthcare insurance coverage support, and re-evaluation of its insurance eligibility through price adjustments or value demonstration is needed. For reimbursement decision-makers in both countries, adopting LU-AT at current prices may impose pressure on healthcare insurance funds due to its high incremental costs relative to modest QALY gains. The model in this study provides a baseline for re-evaluating cost-effectiveness post-price negotiations in both China and the United States, supporting evidence-based deliberations on its inclusion in insurance formularies after achieving optimized pricing. Ultimately, this study strengthens the link between clinical evidence and healthcare insurance policies in China and the United States, aiding in balancing therapeutic innovation, patient access, and the sustainable utilization of healthcare resources.

To the best of our knowledge, this is the first study to evaluate the efficacy of lurbinectedin combined with atezolizumab versus atezolizumab from the perspective of China’s and the United States’ healthcare system, providing up-to-date clinical evidence. This analysis offers significant reference value for China and the United States. While LU-AT was not deemed cost-effective compared to AT, it demonstrated a notable improvement in QALYs for patients with ES-SCLC (0.88 vs. 0.67 QALYs). However, several limitations of the study should be acknowledged. First, data limitations arose as long-term survival data beyond the clinical trial follow-up period were unavailable. Survival models were used to simulate data beyond the follow-up, potentially introducing bias compared to actual data. The cost-effectiveness analysis will be updated when long-term survival data become accessible. Second, the IMforte trial only included a small cohort of Asian populations such as patients from Taiwan, China, and South Korea. Due to potential ethnic differences across populations, this may have an impact on the study results. Third, Data on second-line treatment were derived solely from the IMforte trial and published literature, which may not fully reflect real-world clinical practices. Fourth, the model only accounted for grade 3 or higher adverse events with an incidence greater than 5%. However, sensitivity analysis indicated that variations in the probability of severe adverse events did not significantly affect the results. Despite these limitations, the study provides valuable insights for decision-makers considering lurbinectedin combined with atezolizumab as a first-line treatment for ES-SCLC in China and the United States.

## 5 Conclusion

This study is the first to assess the cost-effectiveness of LU-AT using recent clinical trial data from the perspective of China’s and the United States’ healthcare system. Our findings indicate that, as a first-line treatment for ES-SCLC, LU-AT is not cost-effective compared to AT.

## Data Availability

The original contributions presented in the study are included in the article/**Supplementary Material**. Further inquiries can be directed to the corresponding author.
