# Supplementary material for: Cost-effectiveness analysis of lurbinectedin plus atezolizumab as first-line treatment for extensive-stage small-cell lung cancer
Source: Front Immunol. 2025 Sep 23;16:1658740. doi: 10.3389/fimmu.2025.1658740 (PMC12500677; doi:10.3389/fimmu.2025.1658740)
Supplement: Supplementary file 1 [file DataSheet1.docx]

Supplementary Material

**Cost-effectiveness Analysis of Lurbinectedin plus Atezolizumab as First-line Treatment for Extensive-stage Small-cell Lung Cancer**

**1. Supplementary Table A. CHEERS 2022 Checklist.**

**2. Supplementary Table B. Comparison of survival models.**

**3. Supplementary Figure 1. Results of the survival curve fit the LU-AT and AT group.**

**1.** **Supplementary Table A. CHEERS 2022 Checklist.**

| **Topic** | **No.** | **Item** | **Reported** |
| --- | --- | --- | --- |
| **Title** |  |  |  |
|  | 1 | Identify the study as an economic evaluation and specify the interventions being compared. | Yes |
| **Abstract** |  |  |  |
|  | 2 | Provide a structured summary that highlights context, key methods, results, and alternative analyses. | Yes |
| **Introduction** |  |  |  |
| **Background and objectives** | 3 | Give the context for the study, the study question, and its practical relevance for decision making in policy or practice. | Yes |
| **Methods** |  |  |  |
| **Health economic analysis plan** | 4 | Indicate whether a health economic analysis plan was developed and where available. | Yes |
| **Study population** | 5 | Describe characteristics of the study population (such as age range, demographics, socioeconomic, or clinical characteristics). | Yes |
| **Setting and location** | 6 | Provide relevant contextual information that may influence findings. | Yes |
| **Comparators** | 7 | Describe the interventions or strategies being compared and why chosen. | Yes |
| **Perspective** | 8 | State the perspective(s) adopted by the study and why chosen. | Yes |
| **Time horizon** | 9 | State the time horizon for the study and why appropriate. | Yes |
| **Discount rate** | 10 | Report the discount rate(s) and reason chosen. | Yes |
| **Selection of outcomes** | 11 | Describe what outcomes were used as the measure(s) of benefit(s) and harm(s). | Yes |
| **Measurement of outcomes** | 12 | Describe how outcomes used to capture benefit(s) and harm(s) were measured. | Yes |
| **Valuation of outcomes** | 13 | Describe the population and methods used to measure and value outcomes. | Yes |
| **Measurement and valuation of resources and costs** | 14 | Describe how costs were valued. | Yes |
| **Currency, price date, and conversion** | 15 | Report the dates of the estimated resource quantities and unit costs, plus the currency and year of conversion. | Yes |
| **Rationale and description of model** | 16 | If modelling is used, describe in detail and why used. Report if the model is publicly available and where it can be accessed. | Yes |
| **Analytics and assumptions** | 17 | Describe any methods for analysing or statistically transforming data, any extrapolation methods, and approaches for validating any model used. | Yes |
| **Characterising heterogeneity** | 18 | Describe any methods used for estimating how the results of the study vary for subgroups. | Not applicable |
| **Characterising distributional effects** | 19 | Describe how impacts are distributed across different individuals or adjustments made to reflect priority populations. | Yes |
| **Characterising uncertainty** | 20 | Describe methods to characterise any sources of uncertainty in the analysis. | Yes |
| **Approach to engagement with patients and others affected by the study** | 21 | Describe any approaches to engage patients or service recipients, the general public, communities, or stakeholders (such as clinicians or payers) in the design of the study. | Not applicable |
| **Results** |  |  |  |
| **Study parameters** | 22 | Report all analytic inputs (such as values, ranges, references) including uncertainty or distributional assumptions. | Yes |
| **Summary of main results** | 23 | Report the mean values for the main categories of costs and outcomes of interest and summarise them in the most appropriate overall measure. | Yes |
| **Effect of uncertainty** | 24 | Describe how uncertainty about analytic judgments, inputs, or projections affect findings. Report the effect of choice of discount rate and time horizon, if applicable. | Yes |
| **Effect of engagement with patients and others affected by the study** | 25 | Report on any difference patient/service recipient, general public, community, or stakeholder involvement made to the approach or findings of the study | Not applicable |
| **Discussion** |  |  |  |
| **Study findings, limitations, generalisability, and current knowledge** | 26 | Report key findings, limitations, ethical or equity considerations not captured, and how these could affect patients, policy, or practice. | Yes |
| **Other relevant information** |  |  |  |
| **Source of funding** | 27 | Describe how the study was funded and any role of the funder in the identification, design, conduct, and reporting of the analysis | Yes |
| **Conflicts of interest** | 28 | Report authors conflicts of interest according to journal or International Committee of Medical Journal Editors requirements. | Yes |

**2.** **Supplementary Table B. Comparison of survival models.**

|  | AIC | | BIC | |
| --- | --- | --- | --- | --- |
|  | LU-AT group | AT group | LU-AT group | AT group |
| OS |  |  |  |  |
| Exponential | 918.8790 | 1026.861 | 922.3679 | 1030,346 |
| Gamma | 899.4321 | 1002.904 | 906.4100 | 1009.874 |
| Gen.F | 901.1526 | 1005.171 | 915.1083 | 1019.110 |
| Gen.gamma | 900.5572 | 1003.165 | 911.0240 | 1013.619 |
| Gompertz | 903.3544 | 1017.000 | 910.3323 | 1023.970 |
| Weibull | 898.5734 | 1005.523 | 905.5513 | 1012.492 |
| Log-logistic | 901.1574 | 1001.814 | 908.1352 | 1008.784 |
| Log-normal | 911.8814 | 1002.688 | 918.8592 | 1009.658 |
| PFS |  |  |  |  |
| Exponential | 1060.228 | 1000.0717 | 1063.717 | 1003.5565 |
| Gamma | 1046.735 | 996.9482 | 1053.713 | 1003.9178 |
| Gen.F | 1032.646 | 853.7570 | 1046.602 | 867.6961 |
| Gen.gamma | 1030.637 | 907.3150 | 1041.104 | 917.7694 |
| Gompertz | 1061.064 | 984.5390 | 1068.042 | 991.5086 |
| Weibull | 1051.973 | 1001.9746 | 1058.951 | 1008.9442 |
| Log-logistic | 1037.284 | 924.4286 | 1044.262 | 931.3982 |
| Log-normal | 1029.853 | 928.5042 | 1036.831 | 935.4738 |

AIC, Akaike information criterion; BIC, Bayesian information criterion; LU-AT, Lurbinectedin-Atezolizumab group; AT, Atezolizumab group; OS, overall survival; PFS, progression-free survival

**3.** **Supplementary** **Figure 1. Results of the survival curve fit the LU-AT and AT group.**


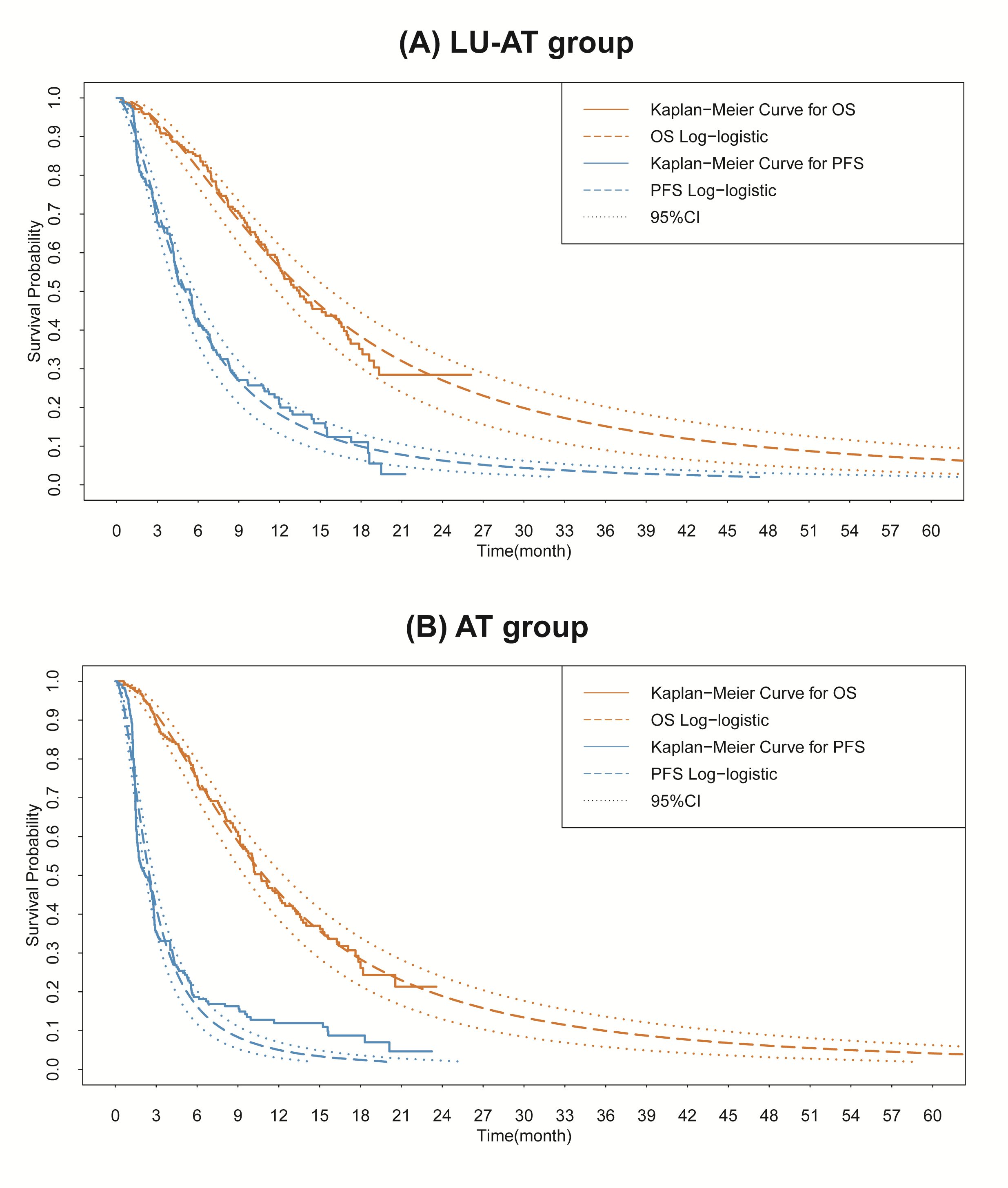


95%CI: 95% confidence interval; LU-AT, Lurbinectedin-Atezolizumab group; AT, Atezolizumab group; OS, overall survival; PFS, progression-free survival.
